# Comparative phosphoproteomic analysis reveals signaling networks regulating monopolar and bipolar cytokinesis

**DOI:** 10.1038/s41598-018-20231-5

**Published:** 2018-02-02

**Authors:** Özge Karayel, Erdem Şanal, Sven H. Giese, Zeynep Cansu Üretmen Kagıalı, Ayşe Nur Polat, Chi-Kuo Hu, Bernhard Y. Renard, Nurcan Tuncbag, Nurhan Özlü

**Affiliations:** 1 Department of Molecular Biology and Genetics, Koç University, Istanbul, Turkey; 2 Bioinformatics Division (MF1), Robert Koch Institute, Berlin, Germany; 3 Chair of Bioanalytics, Institute of Biotechnology, Technische Universität Berlin, Berlin, Germany; 4 Department of Genetics, Stanford University, School of Medicine, CA, USA; 5 Graduate School of Informatics, Department of Health Informatics, METU, Ankara, Turkey; 6 Cancer Systems Biology Laboratory (CanSyL), METU, Ankara, Turkey; 7 Koç University Research Center for Translational Medicine (KUTTAM), Istanbul, Turkey

## Abstract

The successful completion of cytokinesis requires the coordinated activities of diverse cellular components including membranes, cytoskeletal elements and chromosomes that together form partly redundant pathways, depending on the cell type. The biochemical analysis of this process is challenging due to its dynamic and rapid nature. Here, we systematically compared monopolar and bipolar cytokinesis and demonstrated that monopolar cytokinesis is a good surrogate for cytokinesis and it is a well-suited system for global biochemical analysis in mammalian cells. Based on this, we established a phosphoproteomic signature of cytokinesis. More than 10,000 phosphorylation sites were systematically monitored; around 800 of those were up-regulated during cytokinesis. Reconstructing the kinase-substrate interaction network revealed 31 potentially active kinases during cytokinesis. The kinase-substrate network connects proteins between cytoskeleton, membrane and cell cycle machinery. We also found consensus motifs of phosphorylation sites that can serve as biochemical markers specific to cytokinesis. Beyond the kinase-substrate network, our reconstructed signaling network suggests that combination of sumoylation and phosphorylation may regulate monopolar cytokinesis specific signaling pathways. Our analysis provides a systematic approach to the comparison of different cytokinesis types to reveal alternative ways and a global overview, in which conserved genes work together and organize chromatin and cytoplasm during cytokinesis.

## Introduction

Many components of the cell division mechanism are ancient and evolutionarily conserved across eukaryotic cells. In comparison to the nuclear division, cytokinesis uses the most diverse cellular pathways^1^. Cytokinesis requires the coordinated action of multiple cellular processes including membrane trafficking, cell adhesion and cell contractility, actin filaments as well as core cell division components such as microtubules and chromosomes^2,3^. The signal to cleave the cell at the correct time and in the correct place is provided by the midzone, a structure consisting of overlapping bundles of microtubules that form between the separated chromosomes during anaphase. Dynamic spindle microtubules get reorganized into the midzone regulated by conserved phospho-regulation pathways, notably the CDK1, Aurora B and PLK1 kinase signaling networks^4–6^. Communication from the midzone to the actomyosin cortex through Rho signaling spatially and temporally regulates the cleavage furrow formation and ensures that the furrow exactly bisects the segregating chromosomes^7^. Similarly, signals from the cleavage furrow to the midzone stabilize the midzone microtubules in a manner depending on the Aurora B kinase activity^8^.

Incorporation of all these diverse cellular components varies in different cell types. For example, cell adhesion systems have been shown to drive cytokinesis independently from myosin in certain cell types^9–11^. Establishing a robust system to analyze redundant biochemical pathways by systematically comparing cytokinesis in different cell types is expected to yield a better understanding of cytokinesis. A major challenge to this goal is the difficulty of synchronizing different mammalian cells in cytokinesis. Synchronization in mitosis, by activating the spindle assembly checkpoint followed by a wash-out of microtubule-damaging drugs, only allows for partial synchronization, since recovery time after spindle damage varies between cells. A previously developed monopolar cytokinesis system, where cells are arrested in monopolar mitosis followed by cytokinesis induction using a CDK inhibitor^12^ yields an excellent synchronization efficiency. This approach makes a biochemical comparison between cell cycle stages and cell types possible. For example, we were previously able to identify even subtle differences in the binding partners of microtubules and Aurora B kinase during the mitosis-to-cytokinesis transition using this approach^6^. Numerous laboratories also take the advantage of this drug induced monopolar cytokinesis assay to study various aspects of cytokinesis^13–21^. However, pharmacological synchronization methods must be assessed with caution.

In this study, we aimed to systematically evaluate the differences between monopolar and normal cytokinesis using quantitative proteomics to provide insight into both the redundant and conservative pathways during cytokinesis. Our systematic comparison reveals specific signaling networks in monopolar and bipolar cytokinesis, which showed that monopolar cytokinesis specific phosphosites have more sumoylation sites in close proximity than the bipolar specific ones. Therefore, combination of sumoylation and phosphorylation may be part of monopolar cytokinesis specific regulatory mechanisms. Overall, the majority of the phosphoproteome is shared between monopolar and bipolar cytokinesis. Our analysis suggests that monopolar cytokinesis is a competent surrogate for bipolar cytokinesis. By taking the advantage of monopolar cytokinesis, we provide the largest cytokinesis phosphoproteome to our knowledge. We systematically analyzed the cytokinesis phosphoproteome and reconstructed the network of the interactions between kinases and phosphoproteins that revealed 31 different kinases active during cytokinesis.

## Results

### Quantitative Proteomic Comparison between Monopolar and Bipolar Cytokinesis

**Figure 1:**
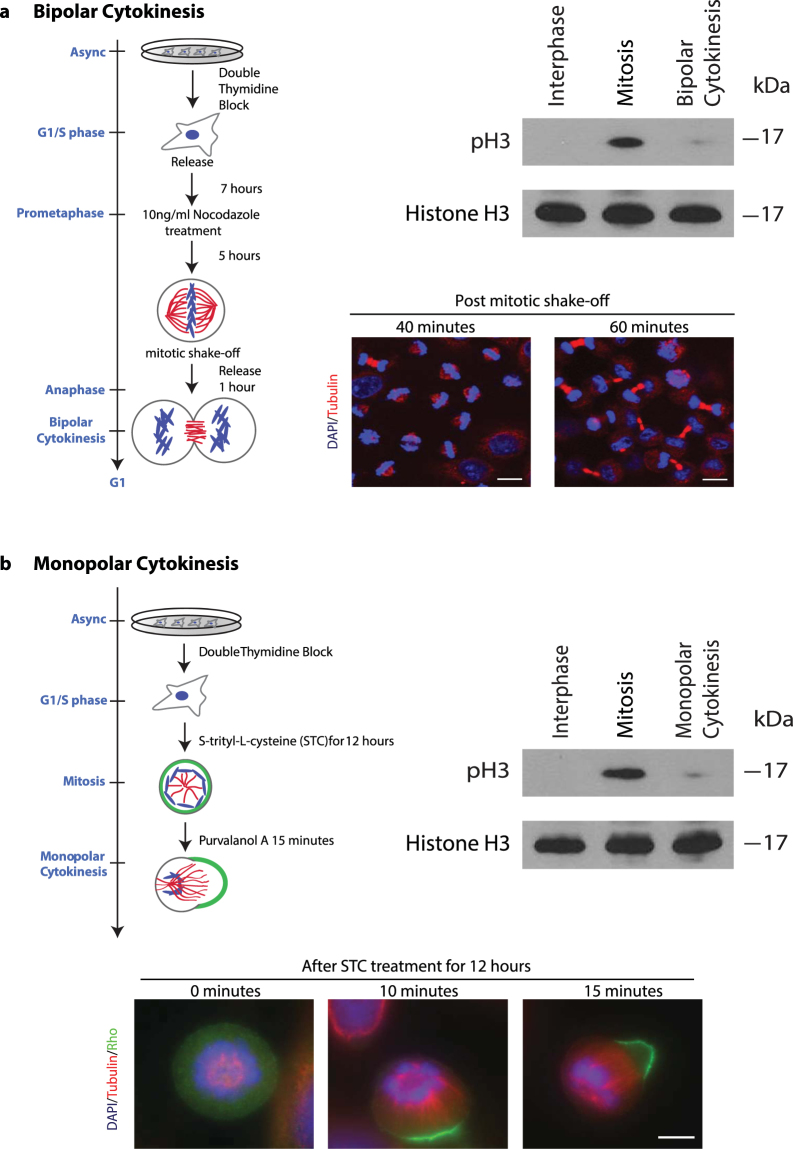
Synchronizations of the bipolar and the monopolar cytokinesis. (**a**) Experimental workflow of the bipolar cytokinesis synchronization. Western blotting against a phosphoserine residue on Histone H3 is shown in the upper panel. The loading control from the same blot using histone H3 antibodies is shown in the bottom panel. Immunostaining of Nocodazole-induced bipolar cytokinesis HeLa cells was probed for tubulin (red) and DAPI (blue) to analyze the cell cycle stages (lower panel) after 40 and 60 minutes from release (scale bar 10 μm). (**b**) Experimental workflow of the monopolar cytokinesis synchronization. Western blotting against the same probes is shown (upper panel). Immunostaining of Purvalanol A-induced monopolar cytokinesis HeLa cells was probed for tubulin (red), Rho (green) and DAPI (blue) after STC treatment for 12 hours (lower panel) (scale bar 5 μm). Full-length blots are presented in Supplementary Fig. 5A, B.

To evaluate the suitability of monopolar cytokinesis as a good biochemical surrogate for normal cytokinesis, we systematically compared monopolar and bipolar cytokinesis cell extracts using stable isotope dimethyl labeling^22^. To synchronize HeLa cells at cytokinesis, we adopted the traditional approach: After double thymidine block, HeLa cells were arrested at G2/M phase with Nocodazole. Following the wash-off step, cells were released into cytokinesis. The synchronized cells were fixed and cytokinesis synchrony was evaluated using immunofluorescence. We optimized the parameters of the protocol for the best synchronization efficiency (Supplementary Figure 1). Accordingly, HeLa cells were arrested in mitosis with 10ng/ml Nocodazole for 5 hours and then released into cytokinesis for 60 minutes. This workflow (Fig. 1a, left), resulted in around 40% of cells at the cytokinesis stage, around 30% were mitotic and the remaining 30% were at the interphase stage. To eliminate the interphase cells, mitotic cells were collected by mechanical shake-off, which improved the cytokinesis synchronization to 60% (Fig. 1a, right bottom). To detect the mitotic cells, we employed the mitotic marker phospho-Histone H3, which appears early in the G2 phase during condensation of chromosomes and disappears again during cytokinesis as mitotic chromosomes start to de-condense^23^. Western blotting against phospho-Histone H3 confirmed that the cytokinesis cells were clearly separated from mitotic cells (Fig. 1a, right top).

For monopolar cytokinesis, HeLa cells were arrested in monopolar mitosis using the kinesin-5 inhibitor S-trityl-L-cysteine (STC) and C-phase was induced by applying the CDK1 inhibitor Purvalanol A for 15–20 minutes (Fig. 1b, top left). As reported earlier^12^, when CDK1 is inhibited, the radial symmetry of microtubules in monopolar mitotic cells is broken and strong polarization begins within minutes after adding Purvalanol A. As microtubules form a focused bundle, the cortex polarizes and mediates the recruitment of contractile ring proteins to the furrow-like cortex where monopolar cytokinesis occurs (Fig. 1b, bottom). Through this method, more than 85% of cells can be synchronized at the cytokinesis cell stage^24^. Similar to bipolar cytokinesis, western blotting analysis further confirmed the reduction of the band corresponding to the mitotic marker phospho-Histone H3 in monopolar cytokinesis (Fig. 1b, right top).

**Figure 2:**
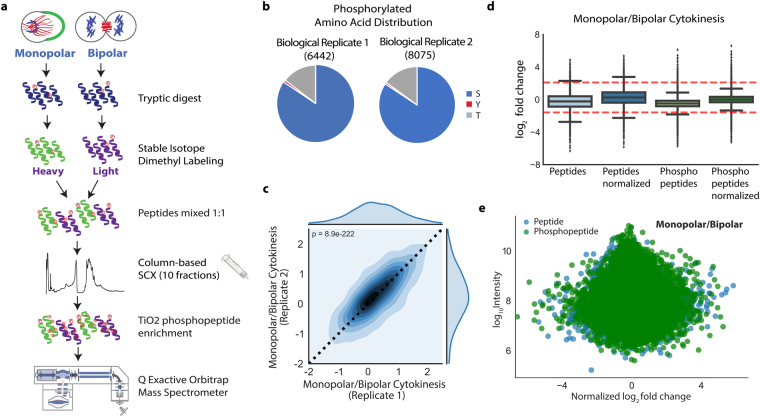
The global phosphorylation analysis of bipolar versus monopolar cytokinesis. (**a**) Experimental workflow of global phosphorylation analysis of bipolar versus monopolar cytokinesis. (**b**) Venn diagrams show the number of phosphosites and their amino acid distribution that are identified in the biological replicates (S: Serine, T: Threonine, Y: Tyrosine). (**c**) The correlation of phosphopeptides between the biological replicates is shown in the plot with a p-value of 8.9e-222. (**d**) The box plot shows the distribution of log_2_ fold changes before and after the normalization for both phosphorylated and all peptides. (**e**) The distribution of phospho- and non-phosphopeptides based on their log_10_ intensities (Y-axis) versus log_2_ fold changes (X-axis) is illustrated. Green represents phosphopeptides and blue represents all other identified peptides.

For a systematic quantitative comparison, we performed tryptic digestion of clarified lysates from cells synchronized in monopolar and bipolar cytokinesis. The resulting peptides were differentially labeled with light and heavy isotopes using stable isotope dimethyl labeling^22^. To eliminate any labeling bias in one biological replicate, heavy monopolar/light bipolar cytokinesis was analyzed and the labels were swapped in the second biological replicate. Two technical replicates were performed for each biological replicate. We combined their differentially labeled peptides in equal amounts and then fractionated the combined peptides using column-based SCX chromatography into 10 fractions. For each fraction, metal-oxide affinity based phosphopeptide enrichment was performed and the sample was analyzed by LC-MS/MS twice. All raw MS data were then processed using Proteome Discoverer software to identify and quantify phosphopeptides. Our experimental workflow is outlined in Fig. 2a.

As a result of all the analyses, 5,467 unique proteins and 68,196 peptides in biological replicate 1 and 5,726 unique proteins and 70,654 peptides in biological replicate 2 were identified with a FDR of 1% for proteins and peptides (Supplementary Table 1). When phosphopeptide enrichment was performed in all SCX fractions, 6,442 and 8,075 phosphopeptides were identified (1% FDR) in biological replicates 1 and 2, respectively. The distribution of all identified phosphorylated residues is shown in Fig. 2b. Figure 2c shows the correlation of phosphopeptides in both biological replicates, with a p-value of 8.91e-222 indicating high reproducibility of our workflow.

The distribution of all peptides and phosphopeptides is shown in a box plot, before and after normalization (Fig. 2d). The red lines in the figures indicate the thresholds of up- and down-regulated phosphopeptides based on the statistical analysis. The distribution of peptides (blue) and phosphopeptides (green) based on their fold-change in monopolar/bipolar conditions is centered around zero (Fig. 2e). Among 5,086 unique common phosphopeptides in both biological replicates, 311 phosphopeptides were significantly up-regulated (monopolar cytokinesis selective) and 130 significantly down-regulated (bipolar cytokinesis selective) (Supplementary Table 2).

The outliers that are unique to one condition are substantially small subgroups. Less than 10% of phosphopeptides were significantly differentially regulated between normal and monopolar cytokinesis. We cannot exclude the possibility that some of the discrepancy is due to differences in the synchronization efficiency between monopolar and bipolar cytokinesis. Nevertheless, the substantial overlap in the phosphorylation patterns of monopolar versus bipolar cytokinesis (Fig. 2) suggests that the monopolar system is well suited as a proxy model for studying cytokinesis biochemistry.

**Figure 3:**
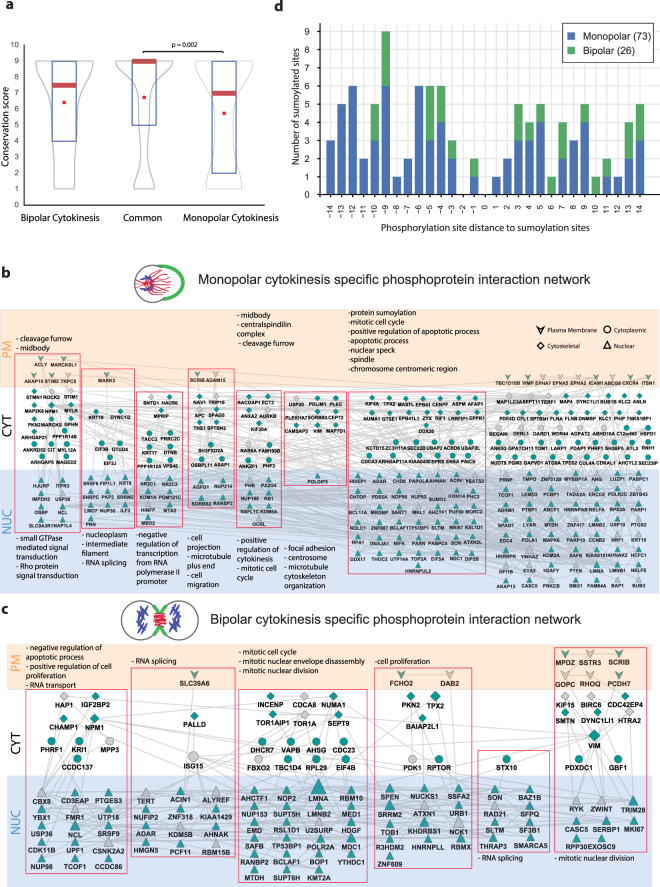
Analysis of monopolar and bipolar cytokinesis selective phosphorylation. (**a**) Conservation scores of the monopolar selective, bipolar selective and shared phosphorylation sites are shown. Median scores of phosphorylation sites found in monopolar cytokinesis and in both are significantly different (Mann-Whitney U test). (**b**,**c**) The reconstructed interaction networks of monopolar selective (**b**) and bipolar selective (**c**) cytokinesis are shown where both phosphorylated proteins (green) and hidden proteins (called Steiner proteins, grey) are represented in a network context. Highly interconnected subnetworks are highlighted in red rectangles and enriched GO Cellular Component and Biological Process terms for each subnetwork are reported. The proteins are organized based on their sub-cellular compartments (plasma membrane, cytoplasm, cytoskeleton and nucleus). The ones with more than one annotated location are only illustrated in one compartment. (**d**) Number of sumoylation sites found within +/−14 amino acids of monopolar and bipolar specific phosphorylation sites. Phosphorylation sites are centered to 0 and the number of sumoylation sites residing upstream and downstream of the phosphorylation sites are reported.

Both cytokinesis models also have unique phosphorylation signatures in addition to common ones. The differences in both cytokinesis models may greatly serve to reveal redundant cytokinesis pathways. We examined to what extent monopolar and bipolar cytokinesis specific phosphosites are evolutionary conserved. The conservation scores of each site, on a scale from 9 (the most conserved) to 1, were calculated using Rate4Site^25^. Strikingly, those phosphopeptides that are common to both monopolar and bipolar cytokinesis are the most conserved ones. The distribution of the phosphopeptides in this category is skewed towards top conservation scores, where the median is 9. The bipolar cytokinesis selective phosphopeptides displayed a distribution of similar conservation scores to slightly lower conservation scores. On the other hand, monopolar cytokinesis selective phosphopeptides are significantly less conserved and have statistically lower conservation scores than the common phosphopeptides that are present in both monopolar and bipolar cytokinesis (Fig. 3a).

Next, we sought to identify the signaling pathways represented by the bipolar and monopolar cytokinesis-specific phosphopeptides (Fig. 3b,c). The significant pathways that display unique phosphorylation pattern in monopolar cytokinesis are mainly cytokinesis related, such as midbody or cleavage furrow formation and Rho signal transduction, suggesting that monopolar cytokinesis may trigger a redundant cortical signaling that are responsible for its unique furrowing morphology along with canonical pathways (Fig. 3b). Conversely, bipolar cytokinesis-specific phosphosites are related to mitotic nuclear division. This is expected, as there is no chromosome segregation in monopolar cytokinesis (Fig. 3c). We further observed that there are slightly more predicted CDK phosphosites left in bipolar cytokinesis (%9) than monopolar cytokinesis (%7). This may be the result of more pronounced regulation of the phosphorylation status of CDK1 substrates by Purvalanol A in monopolar cytokinesis (Supplementary Table 2). Importantly, another post-translational modification, protein sumoylation, is significantly enriched in monopolar cytokinesis which centers around SUMO2 in the reconstructed network (Fig. 3b). SUMO2/3 modification is implicated in chromosome segregation and mitotic exit^26,27^. SUMO2 modification and monopolar specific phosphorylation cross talks may have a unique role in monopolar cytokinesis. To tackle this, we used a comprehensive sumoylation database that is compiled from multiple proteomic studies^28^. We tested whether the monopolar specific phosphopeptides are exclusively sumoylated. Strikingly, when phosphorylation sites are centered to 0 and the number of sumoylation sites within +/−14 amino acids of monopolar and bipolar specific phosphorylation sites are visualized, we observed a significant difference between monopolar and bipolar cytokinesis. Monopolar cytokinesis specific phosphorylations have 73 sumoylation sites near the phosphorylation site, whereas bipolar cytokinesis specific phosphorylations have only 26 sumoylation sites (Fig. 3d, Supplementary Table 2). Monopolar specific phosphorylations in combination with sumoylations may play an important regulatory role in monopolar furrow formation.

### Monitoring Protein Phosphorylation During Cytokinesis

**Figure 4:**
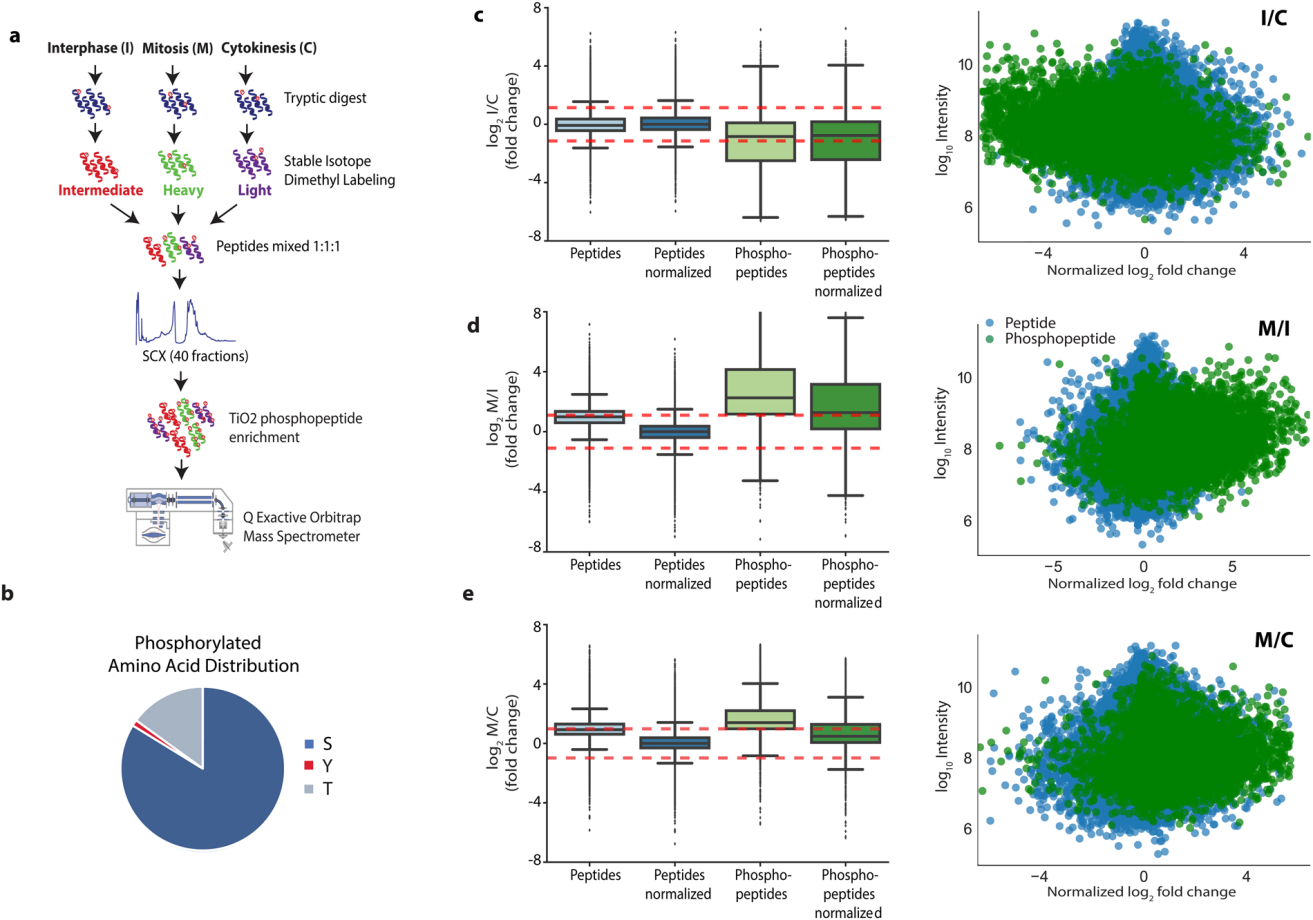
Global analysis of cell cycle phosphorylation patterns. (**a**) Overall experimental workflow. (**b**) The pie chart showing the distribution of phosphorylated amino acids. (**c**–**e**) Box plot of the distribution of log_2_ fold changes before and after normalization for both phosphorylated and all peptides (left). The distribution of phospho- and non-phosphopeptides based on their log_10_ intensities (Y-axis) versus log_2_ fold changes (X-axis) are shown (right) for interphase versus cytokinesis (**c**), for mitosis versus interphase (**d**) and for mitosis versus cytokinesis (**e**).

Because monopolar cytokinesis is a good biochemical surrogate for studying phosphorylation events in cytokinesis, we employed the above mentioned synchronization method to globally investigate protein phosphorylation dynamics during cytokinesis. HeLa cells were tightly synchronized in monopolar cytokinesis, interphase and mitosis. After tryptic digestion, the resulting peptides were labeled using stable isotope dimethyl labeling protocol as light, intermediate and heavy, respectively, and were combined in equal peptide amounts. Global phosphopeptide analysis was performed by peptide fractionation using SCX-HPLC, followed by phosphopeptide enrichment using titanium dioxide (TiO_2_). All peptide fractions were analyzed on a high-resolution Orbitrap instrument coupled with online nanoscale reverse phase HPLC. The experimental workflow is shown in Fig. 4a. In total, 162 LC-MS/MS runs corresponding to 2,720,880 MS/MS spectra, identified 6,230 proteins, 77,410 peptides and after phosphopeptide enrichment, 14,412 unique phosphopeptides at a 1% false discovery rate (Supplementary Table 3). The majority of phosphosites were serine and/or threonine with few tyrosine phosphorylation sites (Fig. 4b). The Proteome Discoverer software was used to quantify the fold change of each peptide in a pairwise fashion (Mitosis/Cytokinesis, Interphase/Cytokinesis and Mitosis/Interphase). The fold changes distributions of quantified peptides and phosphopeptides at different cell cycle stages are shown in Fig. 4c–e. The density plots for threshold estimation from regular peptides of pairwise comparisons are given in Supplementary Fig. 2. As expected, the distribution of phosphopeptides was notably skewed towards mitosis. Likewise, the phosphopeptides are skewed towards cytokinesis in comparison to interphase implying that phosphorylation events gradually decrease as a cell transits from mitosis to cytokinesis and interphase (Fig. 4c–e).

In total, we identified 9,279 unique phosphorylation sites with at least one ratio in three different cell cycle stage comparisons (Mitosis, Cytokinesis and Interphase) and 5,975 phosphopeptides with at least two ratios (Supplementary Table 4). Based on the PhosphoSitePlus database (http://www.phosphosite.org/)^29^ and recent global cell cycle based phosphoproteomic studies^30–35^, 175 out of 9,279 unique phosphorylation sites have not been reported yet (Supplementary Table 4 and Figure 3a).

**Figure 5:**
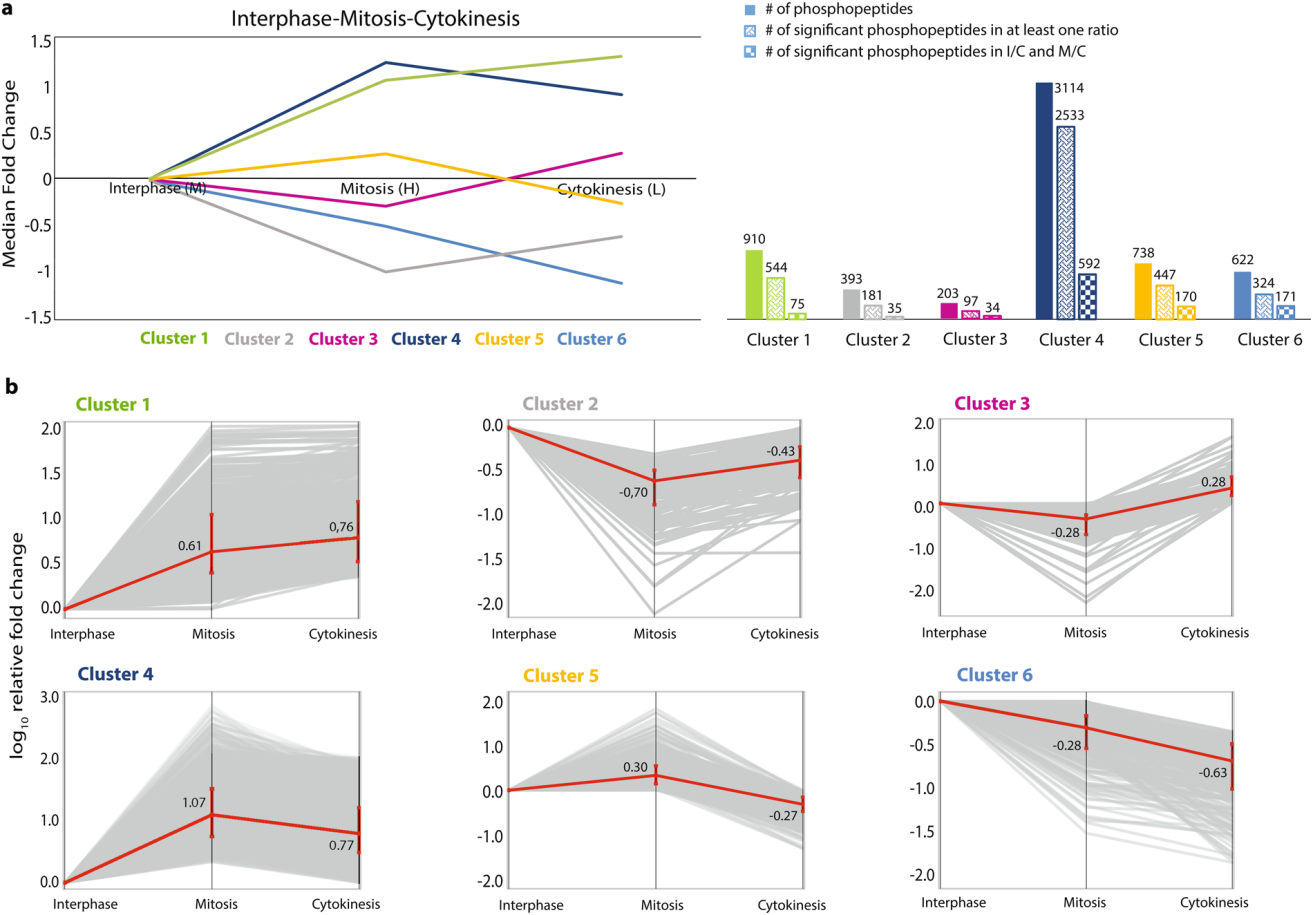
Clustering based phosphorylation analysis of interphase, mitosis and cytokinesis. (**a**) Six main profiles (clusters 1–6) of phosphorylation events are shown in the plot according to their median fold changes through interphase, mitosis and cytokinesis. Median fold changes are in log_10_ scale. The column chart shows the number of the total phosphopeptides and the significantly regulated phosphopeptides in each cluster. M: Medium, H: Heavy, L: Light. (**b**) Phosphopeptides with two pairwise ratios (M/C and I/C), where at least one ratio is significantly different from the rest of its population, were clustered by taking interphase as baseline. The fold change patterns of phosphopeptides (significant in at least one ratio) in clusters are shown with their logarithmic median fold changes (red line) in each cell cycle stage.

Then, we clustered the phosphopeptide abundance profiles for interphase, mitosis and cytokinesis to determine when specific phosphorylation sites peak and are co-regulated during the cell cycle. Six main clusters were identified based on the phosphopeptide dynamics with two ratios throughout the cell cycle (Fig. 5). Cluster 1, 2 and 3 represent the profiles of the phosphopeptides that are up-regulated in cytokinesis compared to mitosis (Fig. 5a), thus dubbed “cytokinesis-selective” (Supplementary Table 4A). Around 1,500 phosphopeptides fell into cytokinesis selective clusters and more than half of those (822) were significantly regulated in at least one cell cycle stage; 144 of those phosphosphopeptides were significantly up-regulated in comparison to both mitosis and interphase (Fig. 5b). Clusters 4 and 5 represent the down-regulated phosphopeptides in cytokinesis compared to mitosis (Supplementary Table 4B). Those ~3,800 phosphosites were mainly dephosphorylated in the transition from mitosis to cytokinesis. Finally, Cluster 6 consists of phosphopeptides with an unusual pattern. These phosphopeptides are down-regulated in both mitosis and cytokinesis relative to interphase.

A recent study by McCloy *et al*.^36^ provides a global overview of the dephosphorylation events during very early stages of mitotic exit. We compared the phosphosites in our study with the sites found in McCloy *et al*. (Supplementary Figure 3b,c). 4,270 phosphosites were common in both datasets and 61% percent of the common phosphosites fall into the clusters 4 and 5 that are mitosis-selective, indeed undergoing dephosphorylation upon mitotic exit (Supplementary Table 4 and Figure 3b). The majority of those sites have a consensus CDK1 and/or CDK2 motifs (Supplementary Table 4 and Figure 3c), which validate our approach.

**Figure 6:**
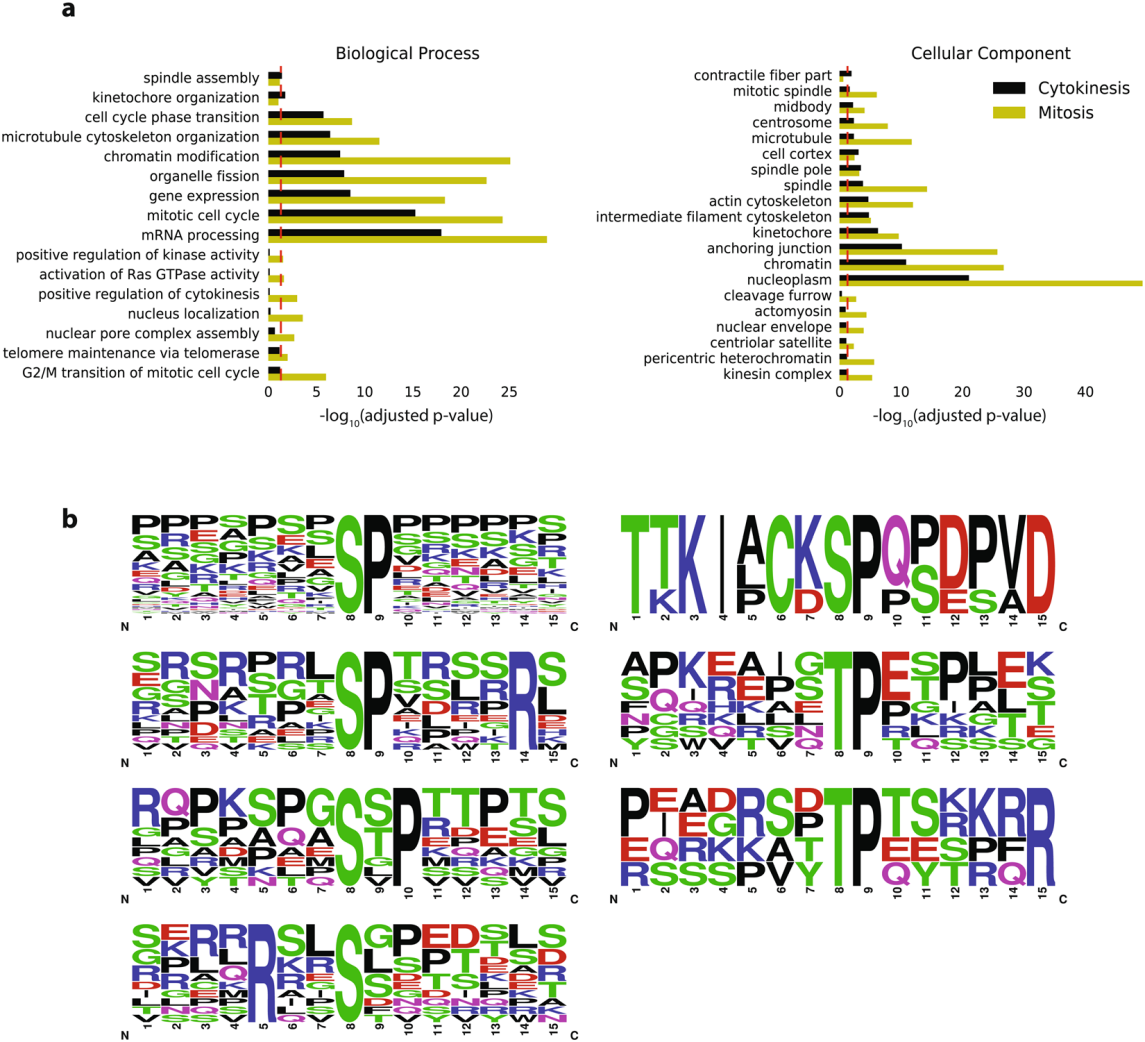
Motif and GO enrichment analysis of cytokinesis selective phosphorylation. (**a**) Comparison of GO biological process and cellular component annotations that are enriched in cytokinesis specific and mitosis specific phosphorylated proteins. Adjusted p-values are calculated via EnrichR (http://amp.pharm.mssm.edu/Enrichr/) and converted into -log_10_. The red line shows the significance threshold, -log_10_ (adjusted p-value) >1.3. (**b**) Enriched motifs of phosphorylation sites that are significantly up-regulated during cytokinesis compared to interphase and mitosis. Motif enrichments are calculated via motif-x (http://motif-x.med.harvard.edu).

GO enrichment analysis of cytokinesis-selective clusters (1,2,3) versus mitotic clusters (4,5,6) did not reveal distinct pathways (Fig. 6a). Proteins, that have both mitosis and cytokinesis specific phosphorylation sites, bring up the importance of the temporal regulation of the phosphorylation and dephosphorylation events during the transition from mitosis to cytokinesis.

We used the motif finder tool motif-x to identify numerically dominant consensus phosphopeptide sequences for significantly regulated phosphopeptides in cytokinesis selective clusters. We found seven distinct motifs in the cytokinesis selective clusters, where pS-P and pT-P were dominant (Fig. 6b), in addition there is a motif from Ki-67 protein, which is discussed below. As expected, pS-P-X-K, which is the most prominent substrate motif for the mitotic master kinase CDK1, was only seen in mitosis selective clusters. It is possible, however, that some of these sites are the result of CDK inhibitor treatment rather than mitosis dependent phosphorylation sites (Supplementary Figure 3C).

### Prediction of Kinase-Substrate Interaction

Next, we searched our data set for the known kinase-substrate relationships annotated in PhosphoSitePlus. Only about 10% of the phosphopeptides (146) from the cytokinesis selective clusters, have at least one known upstream kinase (Supplementary Table 4). The most prominent kinases in this analysis belong to CK1/2, PKA and CDK kinase families.

**Figure 7:**
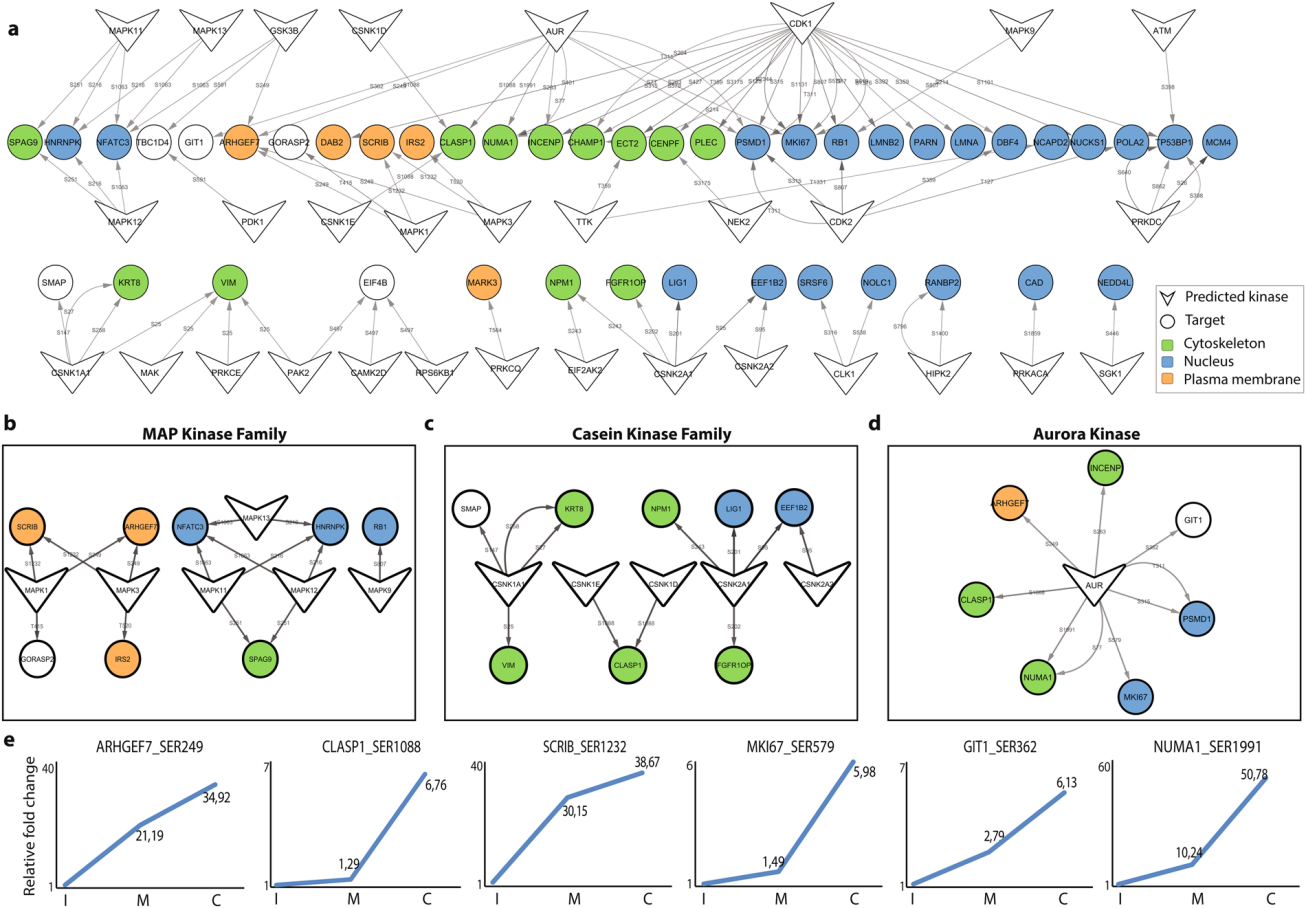
KinomeXplorer based prediction of upstream kinases in cytokinesis enriched clusters. (**a**) The NetworKIN platform is used to predict kinase-substrate interactions in cytokinesis enriched clusters. Only predictions scoring ≥5 are considered and the network is visualized using Cytoscape 3.4.0. The potential substrates are labeled according to their localizations to nucleus (blue), cytoskeleton (green) and plasma membrane (orange). Cellular component annotations for each protein are fetched via the BioServices Python package. Edges are labeled with the position of the significant phosphorylated residue. The cytokinesis selective substrates of MAP Kinases (**b**), Casein Kinases (**c**) and Aurora B kinase (**d**) are shown. (**e**) The representative phosphosites peaking at cytokinesis are shown in the graphs.

We used NetworKIN 3.0 in KinomeXplorer to further predict potential kinase-substrate relationships based on known kinase motifs and protein-protein interactions in the STRINGv10 database^37^. We focused on the significant phosphosites located in cytokinesis-selective clusters to further predict the kinases that play a role in cytokinesis. We identified highly confident interactions between 31 kinases and their substrates (NetworKIN score >5). We propose that these kinases are among the main drivers of cytokinesis (Fig. 7a). MAPK kinase (Fig. 7b) and CK1/2 (Casein) family (Fig. 7c) members represent further highly scored cytokinesis-selective kinases which have a wide range of substrates with indicated phosphosites peaking at cytokinesis. The most striking set of kinase-substrate interactions during cytokinesis is the interaction between Aurora kinase (Fig. 7d) and its substrates including MKI67, CLASP1, NUMA1, INCENP, AGHGEF, GIT1 and PSMD1. Corresponding phosphosites peak in their phosphorylation profile at cytokinesis (Fig. 7e). Another highly scored cytokinesis kinase, TTK (Mps1), has two putative substrates with cytokinesis selective phosphorylation sites, namely: ECT2 and NCAPD2 (Fig. 7a).

### Ki-67 Protein Phosphorylation During Cytokinesis

**Figure 8:**
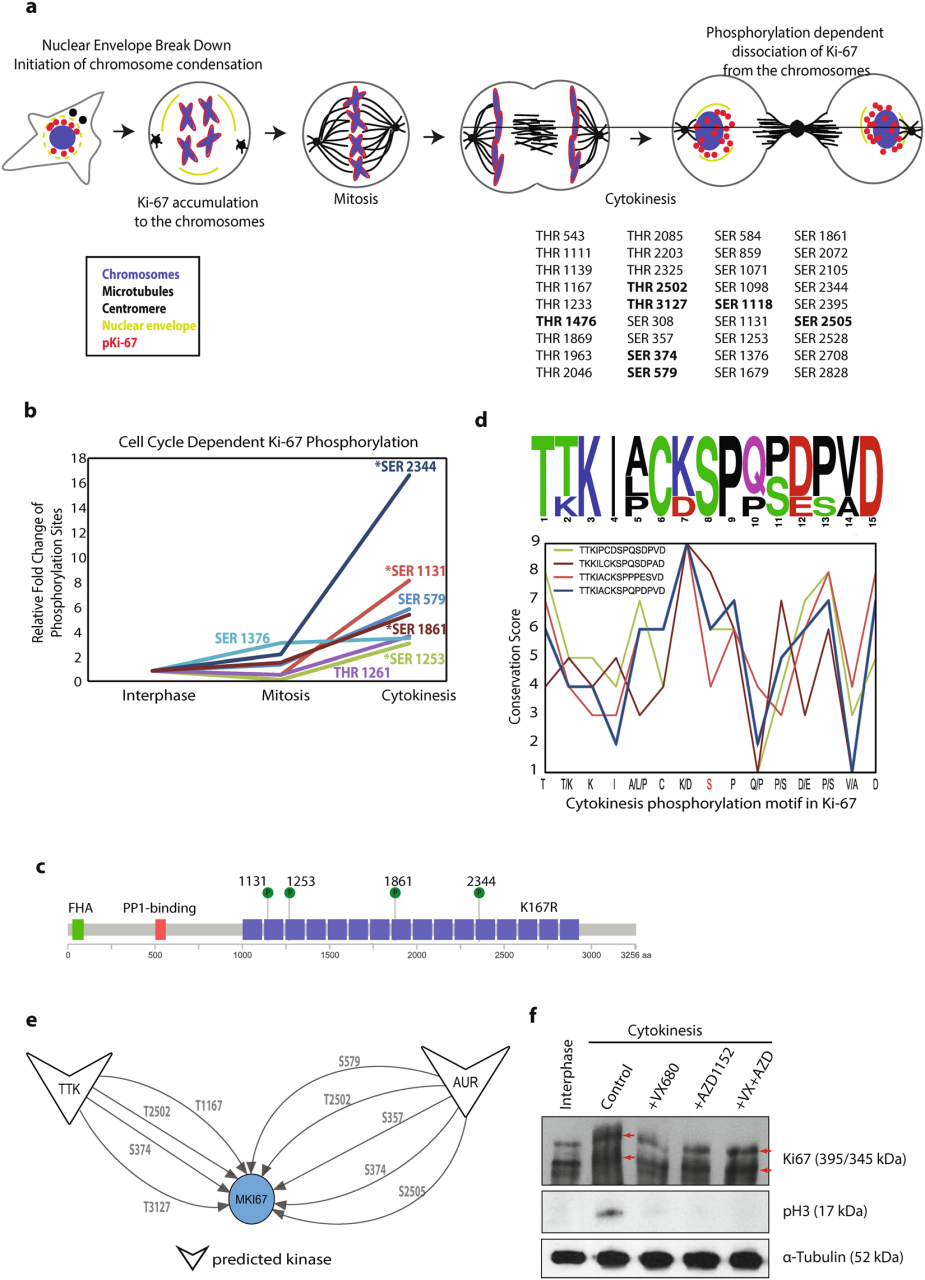
Ki-67 protein phosphorylation during cytokinesis. (**a**) The model of Ki-67 protein phosphorylation during cytokinesis. Synchronization selective phosphosites are also given. (**b**) Cell cycle dependent phosphorylation events of the Ki-67 protein are shown in the graph with fold changes relative to interphase. Asterisk (*) indicates phosphorylation sites in the repeat domain. (**c**) Ki-67 protein domain map. Cytokinesis specific phosphorylation sites with the same motif are shown in the repeat domains. (**d**) The cytokinesis specific phosphorylation motif of Ki-67 with conservation scores for each amino acid. (**e**) KinomeXplorer based predictions scoring ≥5 for upstream kinases of all cytokinesis selective Ki-67 phosphosites are given. (**f**) Western blot analysis of Ki-67 phosphorylation in cytokinesis cells upon Aurora kinase inhibition by VX680 and/or AZD1152 treatment. Full-length blots are presented in Supplementary Figure 5c.

Our global analysis identified Ki-67 (product of the MKI67 gene) as one of the proteins that became highly phosphorylated during cytokinesis. Ki-67 is a proliferation marker that is widely used in basic research and in clinical diagnostics^38^. Ki-67 is a nucleolar protein that localizes to chromosomes during mitosis and it is involved in the reorganization of nucleolar chromatin following mitotic exit^38–41^. As a cell progresses from interphase to mitosis, Ki-67 becomes hyperphosphorylated^42,43^. Overall, our analyses identified 36 phosphorylation sites during cytokinesis in Ki-67 (Fig. 8a). Among them, seven are novel sites that have not been assigned in PhosphoSitePlus database (Fig. 8a, shown in bold).

When we monitored the Ki-67 phosphorylation dynamics in a cell cycle dependent manner, 15 of 36 phosphosites fell into cluster 4 and 5 (mitosis selective clusters, Supplementary Table 5). Seven phosphorylation sites (SER 579, SER 2105, SER 1253, THR 1139, SER 1376, SER 1131 and SER 2344) of Ki-67 were up-regulated only during cytokinesis and fell into cytokinesis enriched clusters (Fig. 8b). Strikingly, four of the cytokinesis selective phosphorylation sites were located in the repeat domains of Ki-67 (Fig. 8c) that are well-conserved across multiple species (Fig. 8d). We hypothesize that the same kinase might be responsible for phosphorylating all these sites in a cytokinesis dependent manner.

Because of the important function of Ki-67 during mitotic exit, we explored the possible kinase-substrate interactions. KinomeXplorer based upstream kinase prediction analysis of cytokinesis selective Ki-67 phosphosites revealed Aurora and TTK (Mps1) as the potential kinases regulating the phosphorylation of the protein during cytokinesis (Fig. 8e). Five potential Ki-67 sites were predicted to be phosphorylated by Aurora kinase (some of which are not specific to cytokinesis). To further examine the Aurora kinase dependent Ki-67 phosphorylation, we arrested HeLa cells in cytokinesis and treated them with Aurora kinase inhibitors, VX680 and AZD1152^44,45^. SDS-PAGE analysis revealed that Aurora kinase inhibition triggered the changes in the electrophoretic mobility of the Ki-67 protein (Fig. 8f). We noticed a dramatic band shift from interphase to cytokinesis, which is in agreement with the hyperphosphorylation of Ki-67 in mitosis. Upon Aurora kinase inhibitor treatment, hyperphosphorylation of Ki-67 is compromised (Fig. 8f). Because Ki-67 is the most commonly used proliferation marker, the phosphosites of Ki-67 that are specific to cytokinesis are very important. Monoclonal antibodies against these phosphosites may greatly serve as biochemical markers for cytokinesis and mitotic exit.

## Discussion

We used quantitative proteomics to analyze how the phosphoproteome changes as the cell progresses into cytokinesis. We identified over 14,000 phosphopeptides and quantified about 10,000 of them in a cell cycle dependent manner. To our knowledge, this is one of the largest datasets that characterizes the phosphorylation events during cytokinesis (C-phase).

In the last decade, the changes in protein phosphorylation during the different stages of the cell cycle have been extensively monitored in multiple phosphoproteomic analyses (reviewed in^26^). Among them, cytokinesis (C-phase) is the shortest (~30–60 minutes)^46^ and the least studied cell cycle stage^26^. The major challenge in cytokinesis is the difficulty of obtaining a homogenous cell population due to the lack of the robust synchronization methods. As a cell enters into cytokinesis, protein phosphorylation may rapidly occur and reach its maximum level followed by a rapid dephosphorylation stage. Tracing such dynamic phosphorylation levels of the sites that peak in cytokinesis in heterogonous cell populations is therefore not feasible. Synchronizing cells in monopolar cytokinesis overcomes the problem^12^. All the characteristic changes of cytokinesis also take place in monopolar cytokinesis; e.g., bundling of midzone microtubules, accumulation of contractile ring effectors to the furrow-like cortex and initiation of cytokinesis specific protein-protein and protein-microtubule interactions^6,12,16^. However, the pharmacological perturbation in the monopolar cytokinesis system raises some concerns over the physiological relevance of the identified changes. In this work, we systematically investigated to what extent monopolar cytokinesis mimics the biochemistry of cytokinesis. Our systematic comparison revealed that the phosphorylation pattern of monopolar and normal bipolar cytokinesis cells highly overlap and that only a small sub-group of phosphorylation sites (~10%) exhibits differential regulation. This result suggests that monopolar cytokinesis is a well-suited system for global biochemical analysis of cytokinesis.

Remarkably, the phosphopeptides that are common in monopolar and bipolar cytokinesis exhibit higher conservation scores than monopolar or bipolar selective phosphosites; albeit the difference was only statistically significant when the common phosphosites are compared to monopolar selective phosphosites. Therefore, further characterization of the common phosphopeptides would provide insight into the core conserved cytokinesis pathways. On the other hand, focusing on the molecular mechanisms of monopolar cytokinesis selective phosphorylations could illuminate the plasticity in cytokinesis pathways. Plasticity in this context means that genes are only used, or required, for certain cell types to divide, or to alternative ways that conserved genes work together and organize chromatin and cytoplasm during division. Our comparative analysis suggests that monopolar specific phosphoproteins are enriched in SUMO-2 modification. SUMO-2 localizes to the chromosome-microtubule interaction sites during anaphase and cytokinesis^27^. Further investigation of cross talks between monopolar cytokinesis specific phosphorylation and sumoylation events may shed light on the underlying molecular mechanism of morphological differences in the furrow formation in monopolar versus bipolar cytokinesis. Extending our comparative cytokinesis approach to different cell types would reveal redundant pathways during cytokinesis.

A previous study^36^ reporting protein dephosphorylation events during the early mitotic exit revealed that a small set of phosphorylation sites get dephosphorylated (10%) at the early mitotic exit. This results suggest that ordered dephosphorylation events by phosphatases regulate mitotic exit and cytokinesis^36^. Our data also supports the gradual de-phosphorylation of mitotic phosphorylation sites and the importance of dephosphorylation in the transition between mitosis and cytokinesis. 13% of mitotic phosphorylation sites get dephosphorylated at cytokinesis. Besides the dephosphorylation events, phosphorylation of around 2% of the sites quantified across three cell-cycle stages (mitosis, cytokinesis, interphase) are up-regulated only in cytokinesis in both biological replicates. Altogether, these results imply that there is cross talk between phosphorylation and dephosphorylation events, and coordination between kinases and phosphatases therefore drives cytokinesis. Consistent with this notion, cytokinesis related proteins possess both mitosis and cytokinesis specific phosphorylation sites which was also demonstrated in multiple other studies^47–49^. The underlying molecular mechanism of interaction between kinases and phosphatases in this complicated circuitry remains to be elucidated.

Which kinases are active during cytokinesis? Our motif-based prediction analysis revealed that 31 kinases including Aurora, Casein and MAP kinase family members, TTK (Mps1), GSKB are most likely active during mitotic exit. Aurora kinase is a master controller of the mitosis-to-cytokinesis transition. Its activity has been previously shown to organize the antiparallel microtubules at the cleavage furrow both *in vivo* and *in vitro*^50,51^. Our analysis identified multiple possible cytokinesis selective Aurora kinase substrates including the CLASP1, NUMA1, MKI67, PSMD1, ARHGEF7, INCENP and GIT1 proteins. CLASP1 (CLIP-associating protein 1), a microtubule plus-end tracking protein^52^, has been identified as a cytokinesis selective microtubule binding protein in our previous study^6^. Phosphorylation of CLASP1 by Aurora kinases promotes microtubule binding activity of CLASP1^6^. Upon its interaction with PRC1 at the onset of anaphase, CLASP1 localizes to the antiparallel microtubules at the central spindle and the PRC1-CLASP1 interaction is required for a stable anti-parallel microtubule organization^53^. Cytokinesis specific phosphorylation of CLASP1 by Aurora B may regulate this interaction. Our analysis predicts cytokinesis selective phosphorylation of ARHGEF7 (Guanine nucleotide exchange factor, PIX) and GIT1 (ARF6 GTPase activating protein GIT) by Aurora kinase. PIX and GIT form a complex, which regulates Rac1 activity^54^. It is plausible that their cytokinesis selective phosphorylation by Aurora plays a role at the spatial and temporal regulation of Rac1 or potentially Rho A during cytokinesis^55–58^ (Supplementary Figure 4).

MAPKs (Mitogen-activated protein kinases) and Casein kinases are other notable active kinase family members identified in cytokinesis in comparison to mitosis and interphase. The literature suggests different functional roles of the activity of both kinase family members during cytokinesis. For example, the MAP kinases ERK1/2 are associated with the midbody and ERK/MAPK activation at the midbody is required for abscission in HeLa cells^59^. The fission yeast ortholog of Casein Kinase1 triggers the signaling module that monitors the mitotic exit checkpoint, and hence controls the timing of cytokinesis^60^. Malik *et al*. also observed increased Casein kinase activity during late mitosis, where the phosphorylation profile of proteins bound to the mitotic spindle was analyzed^61^. Further analysis of potential downstream targets may further shed light on the mechanistic role of the cytokinesis selective kinases.

Another motivation for global biochemical characterization of cytokinesis is to find specific biomarkers that can distinguish between the mitotic and cytokinesis cell stages, which are currently lacking. Multiple anti-mitotic phospho-antibodies such as phospho-Histone H3 are widely used to detect mitotic cells^62^. Extending this to the cytokinesis stage may have great value in assessing cell cycle progression and anti-mitotic cancer chemotherapy. The most striking candidate protein is the proliferation marker Ki-67, which is present in each cell cycle stage (G1, S, G2, Mitosis) of dividing cells but absent in resting cells (G_0_)^38^. Our analysis identified multiple phosphorylation sites of Ki-67 that peak in the cytokinesis phase of the cell cycle. Strikingly, multiple such sites in the K167/Chmadrin repeat domain of Ki-67^63^ show a distinct phosphorylation motif that does not match with any known kinase. Dissecting the molecular mechanism of this cytokinesis specific phosphorylation domain will shed new lights into the responsible kinase and the role of Ki-67 during mitotic exit. Furthermore, motif-based analysis suggested that Aurora and TTK (Mps1) kinases might phosphorylate Ki-67 during cytokinesis. The roles of both kinases in chromosomal organization are well established. We speculate that this signaling network regulates relevant chromosomal reorganizations as cytokinesis progresses. A recent study suggested that Ki-67 is localized on the surface of chromosomes, providing a physical and electrostatic barrier to keep mitotic chromosomes apart during mitosis^39^. Potentially, cytokinesis selective phosphorylation of Ki-67 triggers its dissociation from segregating chromosome peripheries into the nucleolus and temporally controls its unique chromosome segregation function (Fig. 8).

In summary, our study provides the first phosphoproteomic dataset that compares cytokinesis with other cell cycle stages, mitosis and interphase, as well different cytokinesis models; monopolar and bipolar cytokinesis. Beyond the list of phosphorylation sites and proteins, we derive a connected network of interactions between cytoskeleton, membrane and cell cycle machinery. Detailed analysis of these interactions should yield more insights into the biochemical changes during cytokinesis and reorganization of multiple cellular components in the last step of the cell cycle.

## Materials and Methods

### Cell Growth and Synchronization

HeLa cells were grown in Dulbecco’s Modified Eagle’s Medium (Lonza), supplemented with 10% fetal bovine serum (Sigma), 1% penicillin/streptomycin (Sigma) and 1%w/v L-glutamine at 37 °C. For interphase arrest, cells were first treated with 2 mM thymidine (Santa Cruz, 296542 A), released for 8 hours and then treated again with 2 mM thymidine for 17 hours. For mitosis synchronization, cells were treated with 10 µM STC (Sigma, 164739-5G164739) for 12 hours. To induce monopolar cytokinesis, cells were subsequently treated with 30 µM Purvalanol A (Tocris Bioscience, 1580) for 15 minutes and then washed immediately and extensively with PBS.

To induce synchronization in bipolar cytokinesis, following double thymidine blocking, the cells were released for 7 hours, then incubated with 0 ng/ml, 10 ng/ml and 25 ng/ml of Nocodazole (Calbiochem, 487928) for 4 or 5 hours. Next, cells were extensively washed with PBS. Mitotic cells were collected by gentle shaking and incubated for 50 or 60 minutes in the new plate.

### Antibodies and Cell Imaging

For immunostaining or Western blotting, the following primary antibodies were used: anti-phospho-Histone H3 (Ser-10) (32219, Upstate), Histone H3 (9715 S, Cell Signaling), α-Tubulin (3873 S, Cell Signaling), Ki-67 (15580, Abcam) and RhoA (179, Santa Cruz). A Nikon Eclipse 90i Confocal Microscope coupled with the EZ-C1 Imaging Software was used for imaging. For cytokinesis synchronization optimization, at least 10 shots were taken from different spots per slide in 60x magnification and images were quantified using the ImageJ [Wayne Rasband, NIH] software.

#### In Solution Digest and Stable Isotope Dimethyl Labeling

In-solution digestion was performed as described previously^19^. On-column dimethyl labeling was performed as described in^22^. Labeling reagents consisted of 4% (v/v) proper combinations of formaldehyde (CH_2_O, CD_2_O, ^13^CD_2_O), 0.6 M of proper sodium cyanoborohydride (NaBH_3_CN and NaBD_3_CN) (Sigma) in 50 mM NaH_2_PO_4_ (Fisher), and 50 mM Na_2_HPO_4_ (Fisher). The labeled peptides were combined in equal ratios based on their average peptide intensities.

#### Strong Cation Exchange Chromatography and Phosphopeptide Enrichment

Labeled peptide mixes were fractionated using either HyperSep™ SCX Strong Cation Exchange SPE columns or PolySULFOETHYL A 200 × 2.1 mm (PolyLC Inc.) column using an Agilent 1100 HPLC system (Agilent Technologies, Germany). Then, pooled fractions in loading buffer (80% ACN, 6% TFA) were loaded into Titanium dioxide (TiO_2_) (Sachtleben, Germany) packaged micro columns pre-equilibrated with loading buffer, subsequently washed with 50% ACN/0.1% TFA. The bound peptides were eluted by 10% ammonia into 10% formic acid for LC-MS/MS analysis.

#### Data Acquisition

The peptides were subjected to a reversed phase Nano LC-MS/MS (EASY-nLC, Thermo) connected to a Q Exactive quadrupole Orbitrap mass spectrometer (Thermo Fisher Scientific, Bremen). Samples were directly loaded onto an in-house packed 100 μm i.d. × 17 cm C18 column (Reprosil-Gold C18, 5 μm, 200 Å, Dr. Maisch) and separated at 300 nL/min with 75 or 97 minutes linear gradients, increasing from 5% to 40% or 30% acetonitrile in 0.1% formic acid, respectively. Survey spectra were acquired on the Orbitrap with the resolution of 70,000; mass range 350–1500 m/z; automatic gain control (AGC) target 1e^6^; maximum injection time 250 ms in positive mode. The ten most intense ions were selected and then fragmented for the analysis in the Orbitrap. For the full scan of phosphopeptide enriched samples, the resolution and AGC target were set to 35,000 and 3e^6^, respectively. The parameters of MS2 analysis are: resolution 17,500; AGC 2e^5^; maximum injection time 120 ms; normalized collision energy (NCE) 35; charge exclusion unassigned, 1, 7, 8, >8; dynamic exclusion 30, 0 s. The isolation window for MS/MS was 2 m/z and fixed first mass was 100 m/z. For MS2 analysis of phosphopeptide enriched samples, the AGC target and NCE were set to 5e^4^ and 25, respectively. Dynamic exclusion was 20 s and the isolation window for MS/MS was 1,5 m/z. The mass spectrometry data were deposited to the ProteomeXchange Consortium (http://www.proteomexchange.org) via the PRIDE partner repository with the dataset identifier PXD008451.

#### Data Processing

All raw data files were processed and quantified with Proteome Discoverer (PD) (version 1.4, Thermo Scientific). The search was done against the Homo sapiens subset of the Swissprot database version 2014_08 (21,051 sequences) using Mascot (version 2.5.1, Matrix Science, London, UK). Settings for PD search were as following: Mass tolerance of ±20 ppm for precursor masses, and ±0.05 Da for fragment ions, two missed cleavages were allowed, and cysteine carbamidomethylation was set as a fixed modification. Light, medium and heavy dimethylation of peptide N termini and lysine residues; methionine oxidation; and phosphorylation on serine, threonine and tyrosine were set as variable modifications. The identification of the phosphorylation site was done by the phosphoRS 3.0 node of Proteome Discoverer. A probability threshold of at least 0.75 was used for confident phosphosite localization. The 3plex or duplex dimethyl-based quantitation method was chosen in Proteome Discoverer with a mass precision requirement of 2 ppm. We allowed spectra with a maximum of 1 missing channel to be quantified with the integrated Percolator-based filter using a false discovery rate of 0.01.

#### Statistical Significance Analysis

Statistical analysis was performed on the resulting PD peptide tables as described in^19^. The ratio normalization of the phosphopeptides was based on the regular peptides. First, we centered the median intensity of the regular peptides on zero. Then, we corrected the intensity distribution of the phosphopeptides by the same factor as the regular peptides. To derive an empiric fold change (fc) threshold, we fitted a normal distribution to the fc distribution of the regular peptides. The normal distribution was estimated using the median and the median absolute deviation as unbiased estimators for the mean and standard deviation. At alpha = 5%, the 2.5% and 97.5% quantiles of the fitted distribution were further interpreted as significance thresholds for up- or down-regulated peptides. The derived significance thresholds on a log2-scale were defined as follows: monopolar/bipolar cytokinesis: t = |1.0| (replicate 1), t = |0.96| (replicate 2); Mitosis/Cytokinesis: t = |0.98|; Interphase/Cytokinesis: t = |1.14| and Mitosis/Interphase: t = |0.98|.

For the evaluation of the analysis of monopolar versus bipolar cytokinesis with label swaps, we treated the technical replicates as independent trials to assess the significance of the regulation by a binomial probability function. As described previously^19^, to derive a p-value based on the binomial, the number of trials was defined as the number of times, a peptide was quantified; the number of successes was defined as the number of times, a peptide passed the significance threshold; the probability of success was adjusted to the chosen alpha of 5%. Derived p-values were adjusted for multiple testing by Benjamini/Hochberg correction^64^.

#### Conservation Scores

We used BLASTp to search for homologous proteins of phosphoproteins^65^. The E value cutoff was set to 0.0001 and a non-redundant protein database was used. Hypothetical and predicted proteins were removed from the results. The MAFFT-multiple alignment software- was used to align homologous proteins^66^. Evolution rate of each residue was calculated using Rate4Site from the aligned homologous proteins^25^. Evolution rates were scaled to conservation scores between 9 and 1 for each protein. Proteins with intensities lower than 8 in log_10_ scale were excluded. We compared medians of monopolar specific, bipolar specific and shared phosphoproteins’ conservation scores of phosphorylation sites using Mann-Whitney U test.

### Network/Pathway Analysis

The Database for Annotation, Visualization and Integrated Discovery (DAVID) version 6.7 and (https://david.ncifcrf.gov/)^67^ and Enrichr (http://amp.pharm.mssm.edu/Enrichr/)^68^ were used to reveal the enriched annotations for biological function and cellular component. GO subgroups that were significantly enriched with p-values below 0.05 were reported.

NetworKIN 3.0 (as part of KinomeXplorer) was used to generate kinase-substrate networks^69^. Predictions scoring ≥5 were selected. Predicted kinase-substrate interaction networks were visualized using Cytoscape 3.4.0^70^. Cellular component annotations for each protein were fetched via BioServices Python package^71^.

Optimal protein interaction networks were reconstructed by using the Forest module of the Omics Integrator software^72^. The confidence weighted protein-protein interactions in the iRefWeb database^73^ were used as the global interactome and the peptides having significant changes in their phosphorylation status were used as the experimental hits. Then, each peptide was mapped to the corresponding protein. These proteins are called ‘terminals’ in Omics Integrator. If multiple peptides were mapped to the same protein, we took the maximum value of the fold changes. Instead of forcing to connect each terminal directly, the software allows adding intermediate hidden proteins that are not present in the phosphoproteomic set.

#### Cluster Categories and Motif Analysis

Interphase, mitosis and cytokinesis abundance values of each phosphopeptide were calculated from Mitosis/Cytokinesis and Interphase/Cytokinesis ratios by normalizing interphase to 1 and converting the relative ratios to log_10._ Phosphopeptides with two pairwise ratios (Mitosis/Cytokinesis and Interphase/Cytokinesis) where at least one ratio is significantly different from the rest of its population were selected and clustered based on all possible combinations of phosphorylation changes during transition from one phase to the other. First, the phosphorylation status of a site can either be up-regulated or down-regulated during mitosis as compared to interphase. The same applies to the mitosis-cytokinesis transition. Second, the final abundance of a phosphopeptide in cytokinesis can be higher or lower than in interphase. By combining these two criteria, a total of six clusters were defined for the phosphopeptide abundance profiles.

For motif analysis, motif-x^74^ was used. The significance threshold was set to 0.0005 and the motif occurrence limit to 2. The background was set as “IPI human proteome” provided by the motif-x webserver. Weblogo^75^ was used to generate sequence logos.

## Electronic supplementary material

Supplementary Figures

Supplementary Dataset 1

Supplementary Dataset 2

Supplementary Dataset 3

Supplementary Dataset 4

Supplementary Dataset 5
