# Supplementary material for: Comparative phosphoproteomic analysis reveals signaling networks regulating monopolar and bipolar cytokinesis
Source: Sci Rep. 2018 Feb 2;8:2269. doi: 10.1038/s41598-018-20231-5 (PMC5797227; doi:10.1038/s41598-018-20231-5)
Supplement: Supplementary file 1 — Supplementary Figures [file 41598_2018_20231_MOESM1_ESM.pdf]

Comparative phosphoproteomic analysis reveals signaling networks regulating monopolar and bipolar cytokinesis

Özge Karayel[1], Erdem Şanal[1], Sven H. Giese[2,3], Zeynep Cansu Üretmen Kagıalı[1], Ayşe Nur Polat[1], Chi-Kuo Hu[4], Bernhard Y. Renard[2], Nurcan Tuncbag[5,6], Nurhan Özlü*[1,7]

[1]Department of Molecular Biology and Genetics, Koç University, Istanbul, Turkey

[2] Bioinformatics Division (MF1), Robert Koch Institute, Berlin, Germany

[3] Chair of Bioanalytics, Institute of Biotechnology, Technische Universität Berlin, Berlin, Germany

[4] Department of Genetics, Stanford University, School of Medicine, CA, USA

[5] Graduate School of Informatics, Department of Health Informatics, METU, Ankara, Turkey

[6] Cancer Systems Biology Laboratory (CanSyL), METU, Ankara, Turkey

[7] Koç University Research Center for Translational Medicine (KUTTAM), Istanbul Turkey

***To whom correspondence should be addressed:**

Nurhan Özlü, PhD

Department of Molecular Biology and Genetics

Koç University

Istanbul, Turkey

email: nozlu@ku.edu.tr

P: +90 212 338 1571

F: +90 212 338 1559

Running Title: Quantitative Phosphoproteomic Analysis of Cytokinesis

Supplementary Figure 1

Optimization of Bipolar Cytokinesis

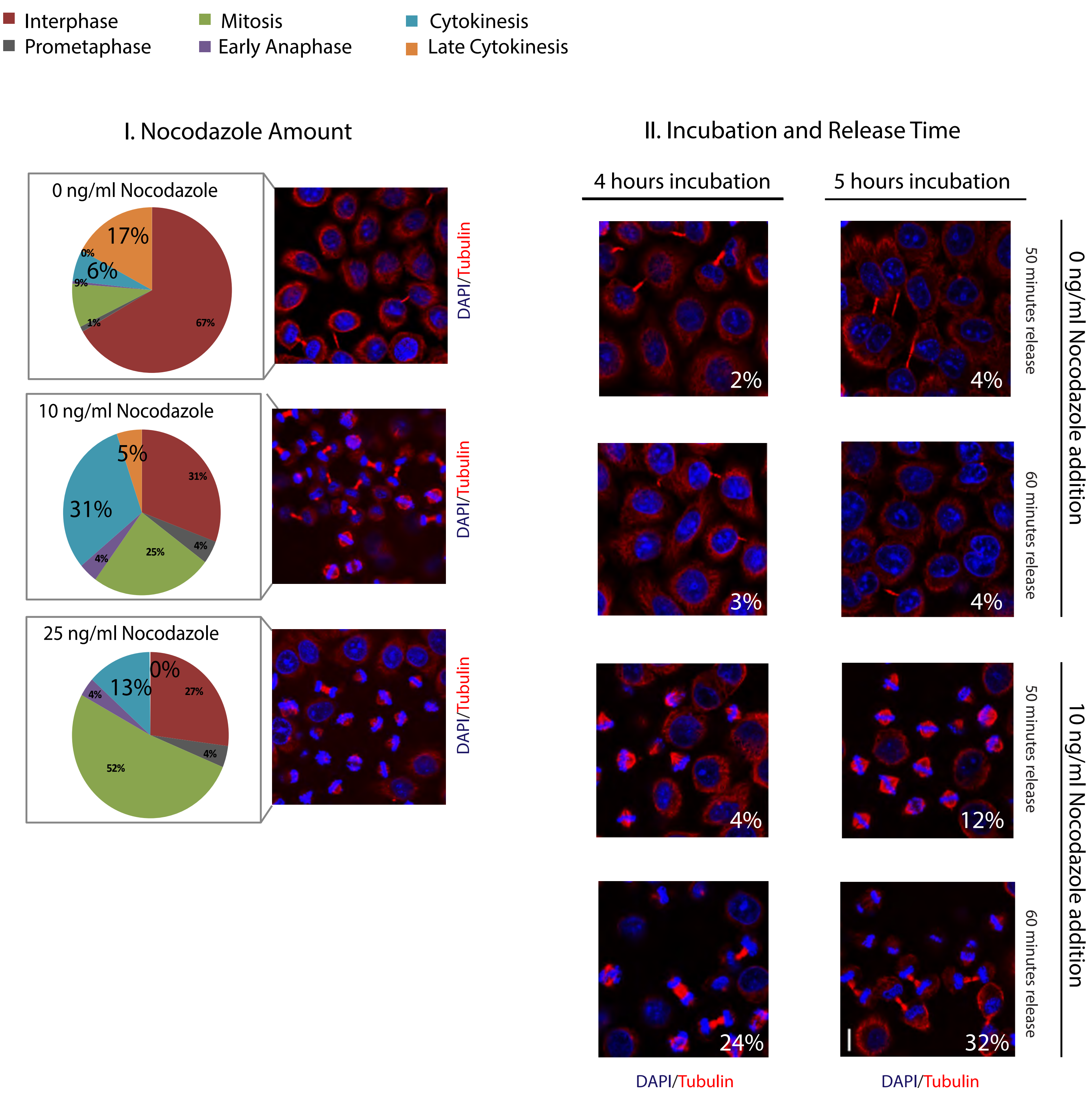

Supplementary Figure 2

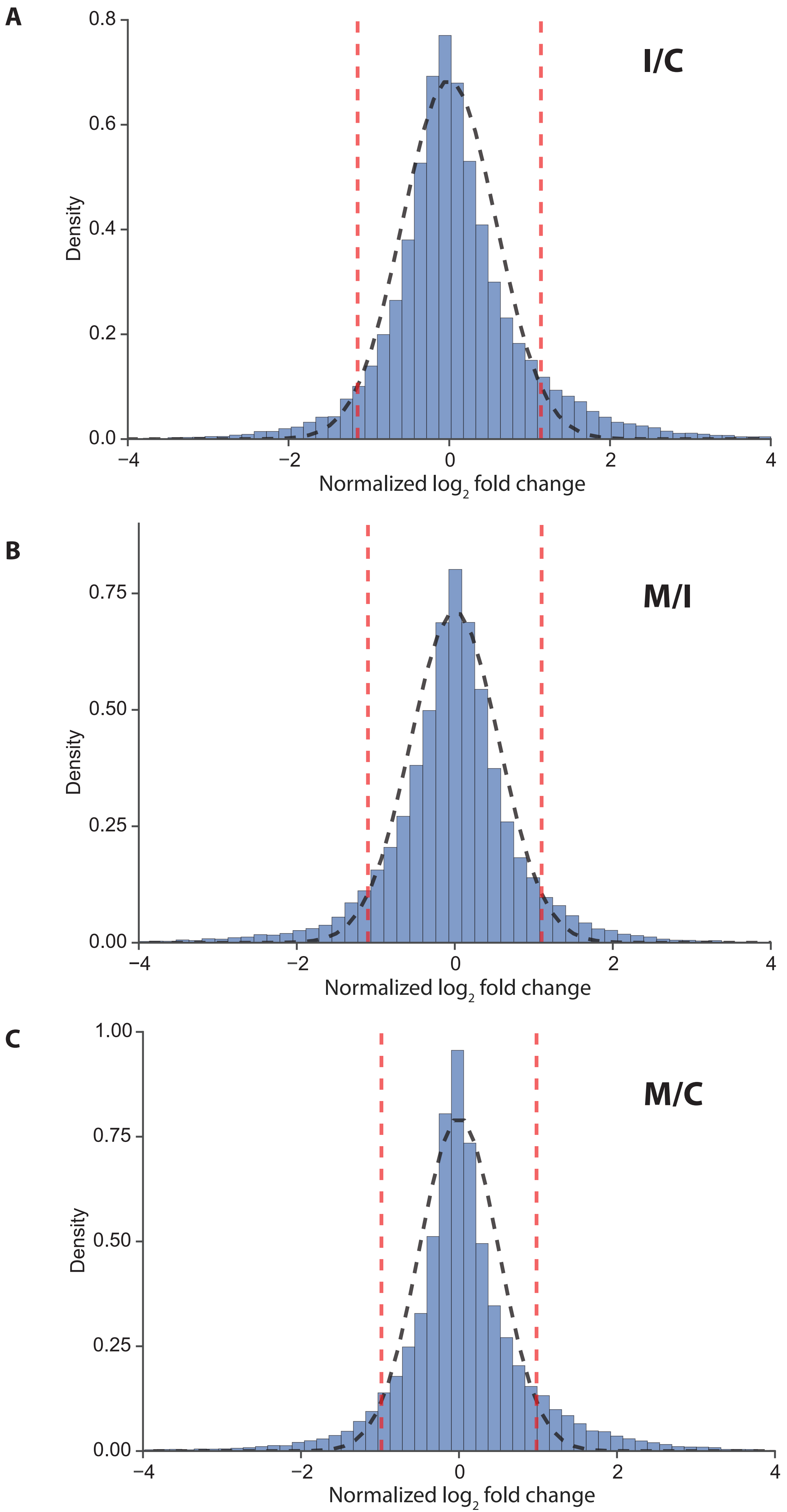

Supplementary Figure 3

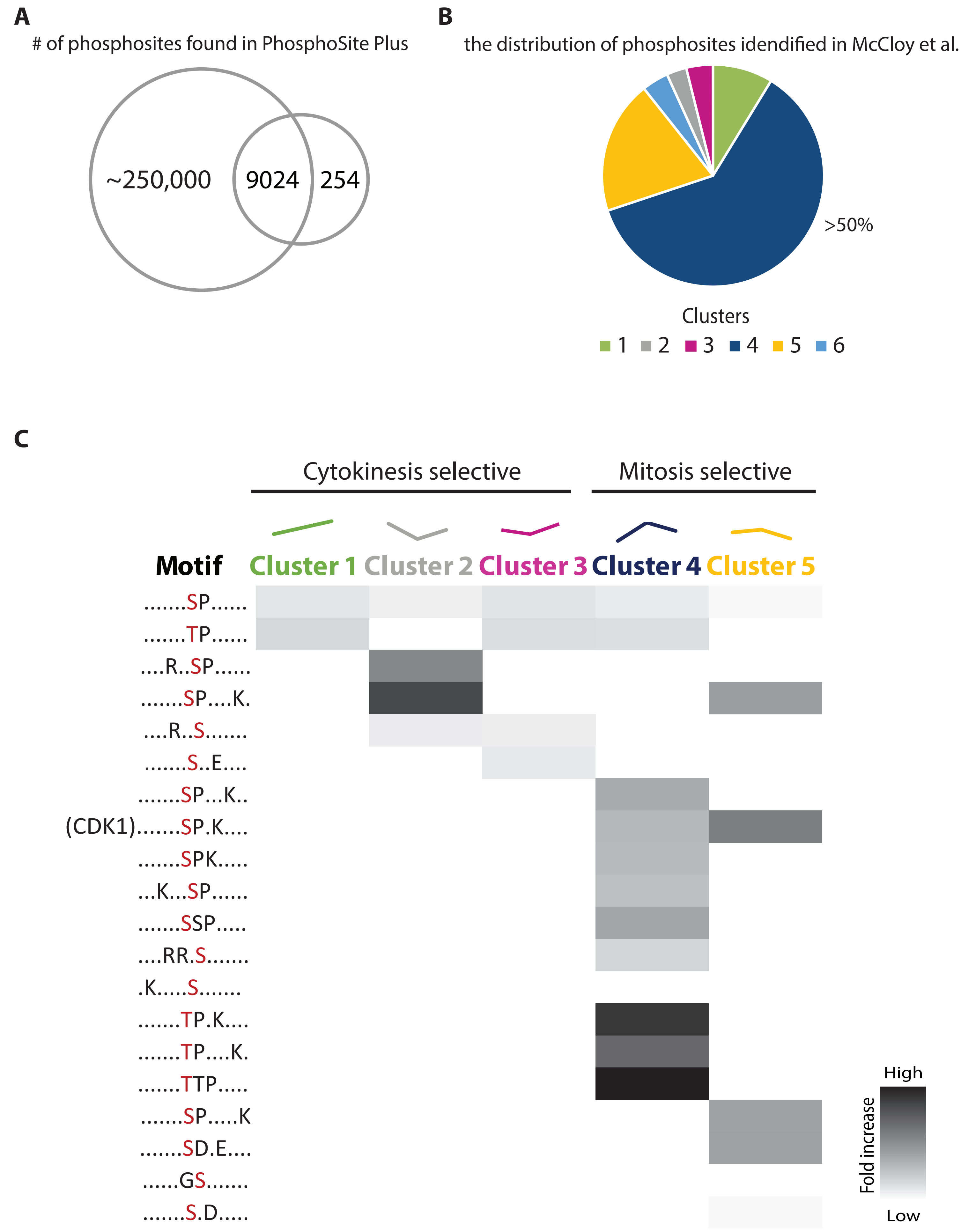

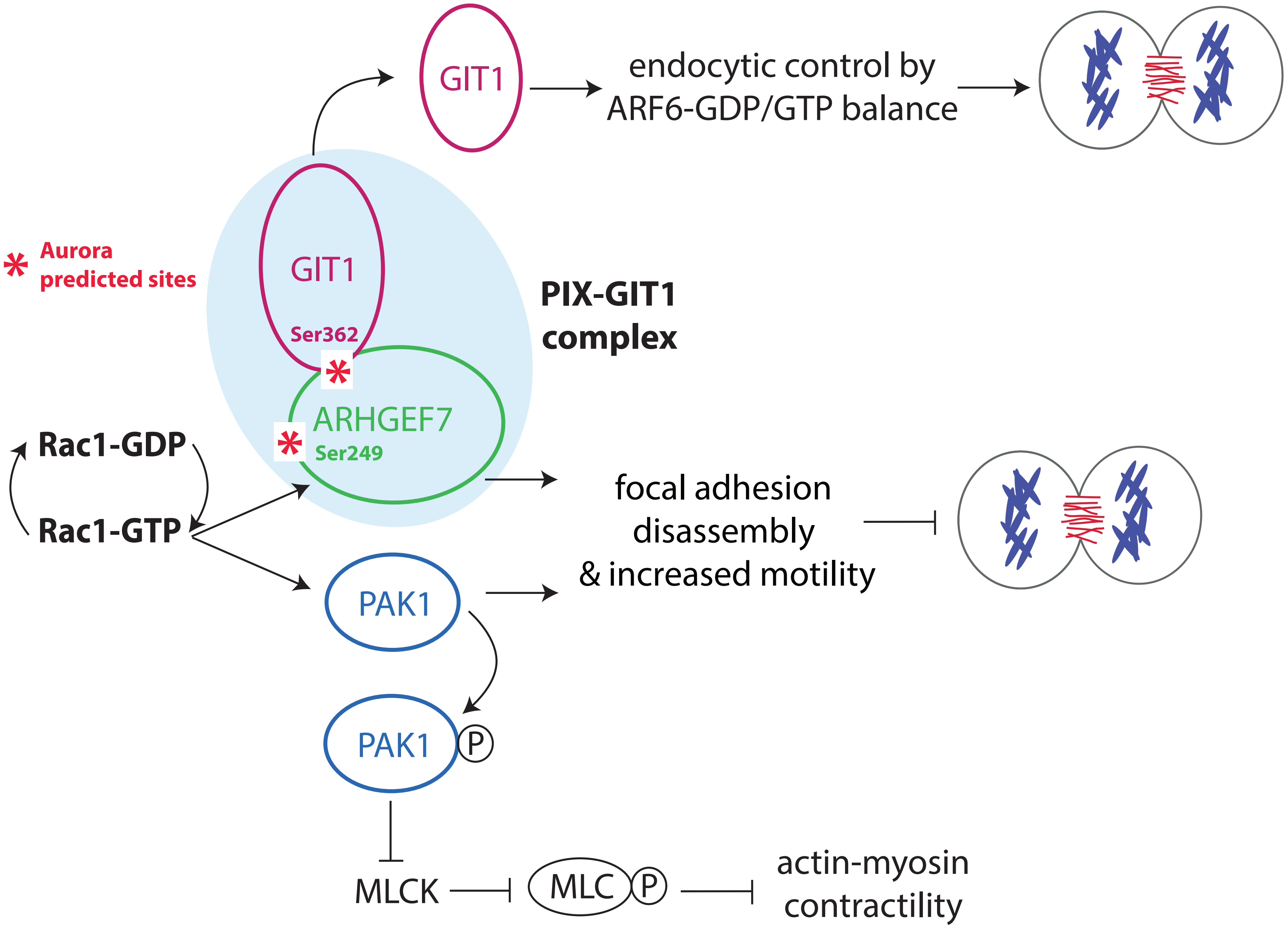
GIT1
endocytic control by
ARF6-GDP/GTP balance
Aurora
predicted sites
GIT1
Ser362
PIX-GIT1
complex
ARHGEF7
Ser249
Rac1-GDP
Rac1-GTP
focal adhesion
disassembly
& increased motility
PAK1
PAK1
P
MLCK
MLC
P
actin-myosin
contractility

Supplementary Figure 5

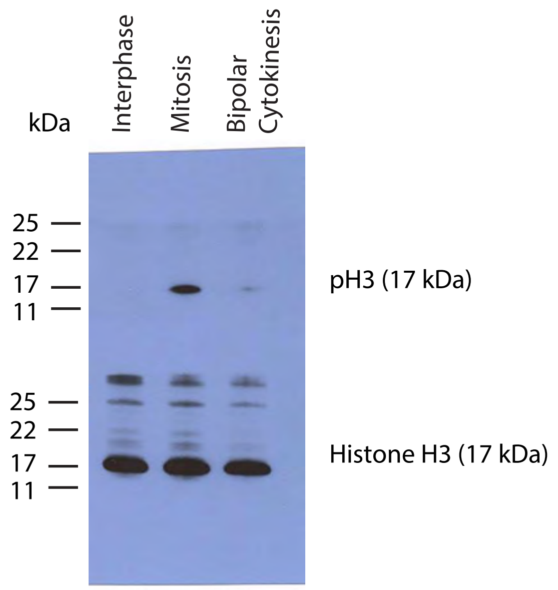

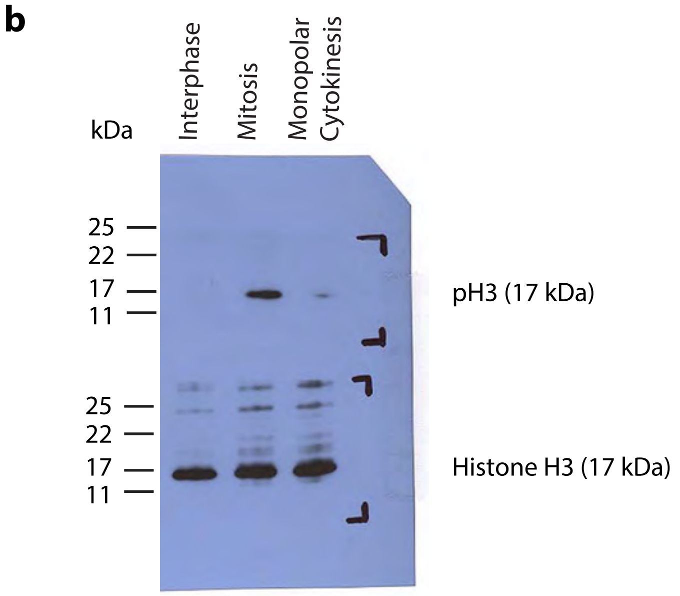

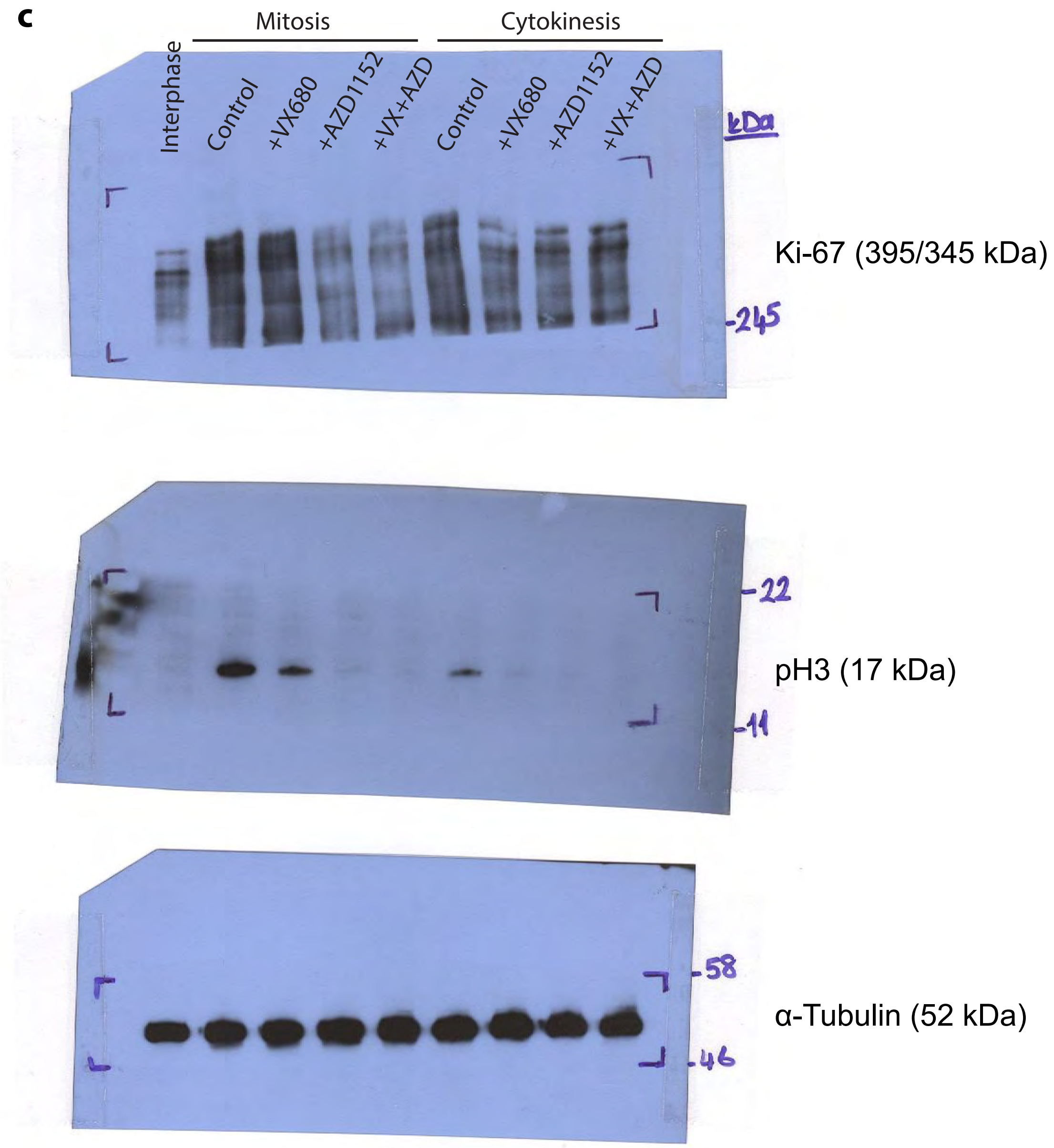

SUPPLEMENTARY FIGURE LEGENDS:

Supplementary Figure 1. The optimization of bipolar cytokinesis arrest using nocodazole. (A) Optimization of nocodazole amount, incubation and release time for bipolar cytokinesis synchronization. Immunostaining of nocodazole induced bipolar cytokinesis HeLa cells was probed for tubulin (red) and DAPI (blue) to distinguish cell cycle stages and further quantify the number of cells. At least 10 images from different spots per slide were taken with 90i Nikon Eclipse Confocal Microscope. The number of cells was counted by ImageJ software (Plugins>Analyze>Cell counter). Total count of cells per sample was normalized to the smallest sample size and percentage of the cell in cytokinesis was determined.

Supplementary Figure 2. Threshold estimation from regular peptides. Density plots for global analysis of cell cycle phosphorylation based on ratio distributions. M: Mitosis, I: Interphase, C: Cytokinesis. The dashed black line shows the normal distribution fit of the logarithmic fold change values. The dashed red vertical lines indicate the corresponding confidence intervals at alpha= 5%, the 2.5% and 97.5% that are used as the significance threshold.

Supplementary Figure 3. Comparison of phosphorylation found in this study to the literature. (A) Venn diagram shows the number of phosphosites which are previously identified in PhosphoSitePlus database[1]. (B) Pie chart shows the cluster based distribution of phosphopeptides which were also identified in[2]. **C.** *De novo* phosphorylation motif analysis in clusters. The sequence windows within 7 amino acids of phosphorylation sites in cytokinesis and mitosis clusters were searched for *de novo* motifs in the motif finder tool motif-x [3].

Supplementary Figure 4. A model for GIT1-PIX complex regulation by phosphorylation during cytokinesis. The positions of phosphorylation sites of PIX (ARHGEF) (Ser249) and GIT1 (Ser362) proteins that match to a characteristic Aurora kinase consensus sequence in our cytokinesis analysis were highlighted with asterisks. PIX and GIT form a complex and GIT1 phosphorylation site is found in the GIT1-PIX interaction domain (SHD domain) whereas PIX phosphorylation site

resides in DH (DBL homology) domain of the protein, which mediates guanine nucleotide exchanges on Rho family GTPases. The complex regulates the Rac1 activity[4-8]. The activated Rac1-PIX-GIT1 complex was shown to activate PAK1 (p21-activated kinase 1), causing downregulation in activity of myosin light chain kinase (MLCK) which normally induces actin-myosin contractility by phosphorylating myosin regulatory light chain (MLC)[9]. In addition to that, ARF6 (ADP-ribosylation factor 6) activity is regulated by GIT1 in endocytic recycling during cytokinesis. ARF6 localizes to the cleavage furrow and midbody of cells during cytokinesis and the overexpression of activated ARF6 at high levels causes cytokinesis defects[10-12].

Supplementary Figure 5. Full-length Western blots. (A) Bipolar cytokinesis analysis presented in Figure 1A. (B) Monopolar cytokinesis analysis presented in Figure 1B. (C) Analysis of Ki-67 phosphorylation in cytokinesis presented in Figure 8F. Ki-67 was analyzed by 3% SDS-PAGE, pH3, Histone H3 and α-Tubulin were analyzed by 12% SDS-PAGE using NEB P7712 marker. For the control of cell synchronizations, pH3 level was analyzed. Histone H3 and α-Tubulin were the loading controls. Western blot images were cropped to improve the conciseness of the data.

REFERENCES:

1 Hornbeck, P. V., Chabra, I., Kornhauser, J. M., Skrzypek, E. & Zhang, B. PhosphoSite: A bioinformatics resource dedicated to physiological protein phosphorylation. *Proteomics* **4**, 1551-1561 (2004).

2 McCloy, R. A. *et al.* Global phosphoproteomic mapping of early mitotic exit in human cells identifies novel substrate dephosphorylation motifs. *Molecular & Cellular Proteomics* **14**, 2194-2212 (2015).

3 Chou, M. F. & Schwartz, D. Biological sequence motif discovery using motif-x. *Curr Protoc Bioinformatics* **Chapter 13**, Unit 13 15-24, doi:10.1002/0471250953.bi1315s35 (2011).

4 Davies, T. & Canman, J. C. Stuck in the middle: Rac, adhesion, and cytokinesis. *J Cell Biol* **198**, 769-771 (2012).

5 Canman, J. C. *et al.* Inhibition of Rac by the GAP activity of centralspindlin is essential for cytokinesis. *Science* **322**, 1543-1546 (2008).

6 Burridge, K. Crosstalk between Rac and Rho. *Science* **283**, 2028-2029 (1999).

7 Bastos, R. N., Penate, X., Bates, M., Hammond, D. & Barr, F. A. CYK4 inhibits Rac1-dependent PAK1 and ARHGEF7 effector pathways during cytokinesis. *J Cell Biol* **198**, 865-880 (2012).

8 Frank, S. R. & Hansen, S. H. in *Seminars in cell & developmental biology.* 234-244 (Elsevier).

9 Zhao, Z.-s. & Manser, E. PAK and other Rho-associated kinases–effectors with surprisingly diverse mechanisms of regulation. *Biochemical Journal* **386**, 201-214 (2005).

10 Chesneau, L. *et al.* An ARF6/Rab35 GTPase cascade for endocytic recycling and successful cytokinesis. *Current Biology* **22**, 147-153 (2012).

11 Schweitzer, J. K. & D'Souza-Schorey, C. A requirement for ARF6 during the completion of cytokinesis. *Experimental cell research* **311**, 74-83 (2005).

12 Schweitzer, J. K. & D'Souza-Schorey, C. Localization and activation of the ARF6 GTPase during cleavage furrow ingression and cytokinesis. *Journal of Biological Chemistry* **277**, 27210-27216 (2002).
